# Sex differences of visceral fat with cardiac structure and function in type 2 diabetes: A cross‐sectional study

**DOI:** 10.1111/1753-0407.70023

**Published:** 2024-11-07

**Authors:** Chen Rui‐hua, Lin Yi, Xu Huan‐bai, Wang Yu‐fan, Peng Yong‐de

**Affiliations:** ^1^ Department of Endocrinology, Shanghai General Hospital Shanghai Jiao Tong University Shanghai China

**Keywords:** diabetes mellitus, type 2, left ventricular ejection fraction (LVEF), left ventricular hypertrophy, visceral adipose tissue

## Abstract

**Background:**

The aim of this study is to analyze the associations among fat distribution, left ventricular (LV) structure, and function in T2DM patients and further assess the sex differences among them.

**Methods:**

Two thousand and one hundred seven T2DM patients were enrolled to this study. Patients' height, weight, BMI, visceral fat area (VFA), baPWV, parameters of cardiac structure and function, and clinical biochemical indicators were measured and collected.

**Results:**

There were significant differences between male and female T2DM patients in age, duration of diabetes, complication ratio of hypertension and dyslipidemia, smoking history, visceral fat, baPWV, and ventricular structure and function (*p* < 0.05). Compared with the Q1 group, female patients in the highest quartile (Q4) of VFA had a decreased LVEF and significantly increased baPWV (*p* < 0.05), whereas no such changes were found in males. The correlation analysis showed that LVEF in male patients was negatively correlated with hypertension history, using of CCBs, GLP‐1RA, lipid‐lowering medications, BMI, WC, WHR, FPG, FC‐P, HbA1c, GA, HOMA‐IR, Cr, and baPWV, while the LVEF in female patients was negatively correlated with VFA, VSR, VFA/BMI, VFA/H^2^, VFA/weight in females (*p* < 0.05). LVMI was positively associated with diabetes duration, age, hypertension history, WC, WHR, VFA, SFA, VFA/BMI, VFA/H^2^, VFA/weight, and baPWV in both males and females. Multivariable‐adjusted linear regression analysis showed that VFA was independently associated with LVEF (*β* = − 0.096, *p* = 0.010), LVMI (*β* = 0.083, *p* = 0.038), and baPWV (*β* = 0.120, *p* = 0.003) in females.

**Conclusions:**

Values of VFA were independently associated with LVEF, LVMI, and baPWV in women, but not in men, in patients with T2DM.

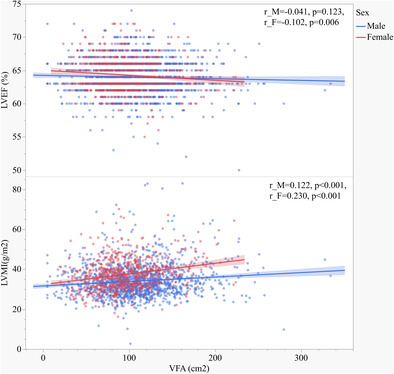

## Graphical abstract

**Highlights**

- Our study suggests sex differences for visceral fat obesity and cardiac hemodynamic parameters.
- VFA is significantly associated with the development of artery stiffness, left ventricular function and deformation in female patients with T2DM.

## 1 INTRODUCTION

The prevalence of type 2 diabetes (T2DM) is increasing rapidly and becoming a global health burden.^1^ China has the largest number of patients with diabetes worldwide, and the number is predicted to rise from 140.9 million in 2021 to 174.4 million by 2045.^2^ Diabetes increases the future risk of heart failure,^3^ coronary heart disease,^4^ peripheral artery disease,^5^ and sudden death.^6^ A growing body of evidence^7^, ^8^ suggests that left ventricular hypertrophy (LVH) is highly prevalent in T2DM patients and may appear before the development of cardiac diseases. The mechanism of the increased risk of LVH in diabetes is the glycosylation of lipids, activation of the renin‐angiotensin system (RAS), increasing of reactive oxygen species, as well as alterations in calcium homeostasis. These disturbances play key roles in extracellular remodeling and hypertrophy of the diabetic heart, thus leading to cardiac dysfunction.^9^

Obesity, generally defined by an excess of body fat causing prejudice to health, is considered to be an independent risk factor for T2DM and cardiovascular disease (CVD).^10^ The growing prevalence of T2DM is largely attributed to the obesity epidemic.^11^, ^12^ However, obesity can no longer be evaluated solely by the body mass index (expressed in kg/m^2^) because it represents a heterogeneous entity. Those who have a normal weight or overweight are at high risk of cardiovascular if they are accompanied with an excess of visceral adipose tissue. On the other hand, individuals who are overweight or obese can nevertheless be at much lower risk than expected if they have more subcutaneous adipose tissue and lower levels of ectopic fat such as less liver fat.^13^ Body fat distribution should be considered rather than crude body weight.^14^

Findings from epidemiological studies have shown that visceral fat is associated with insulin resistance,^15^ structural and functional cardiac remodeling in type 2 diabetes (T2DM),^16^ and metabolic syndrome,^17^ suggesting that obesity‐related diseases are more correlated with visceral fat rather than the accumulation of whole body fat. Considering that the patterns of body fat distribution are different between male and female,^18^ males feature a greater trunk and visceral fat and liver fat compared with females with same age and BMI. The steep rise of T2DM and associated complications goes along with increasing evidence of clinically important sex differences.^19^ To date, there are limited data suggesting the correlations of visceral fat and left ventricular function between male and female.

Based on the above considerations, we designed the present cross‐sectional study to investigate the association of visceral fat with cardiac structure and function in type 2 diabetes patients in a Chinese population in Shanghai, and to further assess the sex differences between them.

## 2 MATERIALS AND METHODS

### 2.1 Study population

This cross‐sectional study was conducted at the National Metabolic Management Center (MMC) of Shanghai General Hospital, a standard nationwide and reproducible platform in China. A total of 2107 patients with T2DM (1385 males and 722 females) were enrolled in this study from April 2018 to May 2022. T2DM was diagnosed according to the 1999 World Health Organization criteria. Exclusion criteria were (1) age <18 years old; (2) BMI < 18.5 kg/m^2^ or ≥40 kg/m^2^; (3) history of myocardial infarction, previous percutaneous coronary intervention, coronary bypass surgery, congenital heart disease, patients with LVEF < 50%, acute coronary syndrome, significant heart valve disease, and significantly abnormal electrocardiogram (ventricular and supraventricular arrhythmias, atrial fibrillation, preexcitation syndrome); (4) recent diabetic acute complications, including diabetic ketoacidosis, lactic acidosis, and hyperosmotic state; (5) type 1 diabetes and gestational diabetes; (6) pregnant or lactating women; and (7) patients with acute or chronic infectious diseases, autoimmune disease, or cancer.

The study was performed in accordance with the second Helsinki declaration and was approved by the Medical Ethics Committee, Shanghai General Hospital, (2017KY209‐5), and was registered on ClinicalTrials.gov (NCTO3811470). All participants provided written informed consents before voluntary participation.

### 2.2 Clinical evaluation

Demographic features including data of age, sex, smoking history, alcoholism, history of diseases, body mass index (BMI), waist circumference (WC), waist‐to‐hip ratio (WHR = neck circumference/hip circumference), neck circumference (NC), systolic blood pressure (SBP), and diastolic blood pressure (DBP) and medication use situations were collected and recorded using a standard beam balance scale. Patients with SBP ≥ 140 mmHg, or DBP ≥ 90 mmHg respectively, or current use of antihypertensive medications were identified as hypertension. Body mass index (BMI) was calculated as weight (kg)/height (m^2^). Medication and supplement use data were obtained for use of lipid‐lowering medications, antidiabetes medications, antihypertensive medications, and antiplatelet medications through questionnaires and pill bottle reviews.

### 2.3 Laboratory methods

Blood samples were obtained from all the patients by venipuncture after an overnight fast (≥8 h). C‐peptide (C‐P) was tested by electrochemiluminescence immunoassay (I2000, Abbott, USA). Fasting blood glucose (FBG), total cholesterol (TC), triglyceride (TG), high‐density cholesterol (HDL‐C), low‐density cholesterol (LDL‐C), serum creatinine (Scr [1 mg/dL = 88.41 μmol/L]), uric acid (UA), and blood urea nitrogen (BUN) were quantified enzymatically (Au5800, Beckman Coulter, USA). HbA1c was measured using high performance liquid chromatography (HPLC) (HA‐8180, ARKRAY, Japan). Estimated glomerular filtration rate (e‐GFR) was calculated by the simplified equation (e‐GFR = 1.86 × Scr^− 1.154^ × age^−0.203×[0.742 if female]^) proposed by the Modification of Diet in Renal Disease (MDRD) Study group.

### 2.4 Assessment of visceral fat area and subcutaneous fat area

Computed tomography, magnetic resonance imaging, and dual‐energy X‐ray absorptiometry can precisely measure entire visceral fat amount of total and regional body composition. However, such methods are expensive and not useful for ubiquitous and frequent use. Bioelectrical impedance analysis (BIA) is a kind of simple, noninvasive, and cost‐effective measurement. Accumulating evidence has confirmed that VFA measured by the dual bioelectrical impedance analyzer was a kind of simple method. It is more cost‐effective and noninvasive than the above methods for estimating the VFA in type 2 diabetes.^20^, ^21^ Visceral fat area (cm^2^) and subcutaneous fat area (SFA) (cm^2^) were measured at the umbilical level by a dual bioelectrical impedance analyzer (Omron HDS‐2000 DUALSCAN, Omron Healthcare Co, Kyoto, Japan). V/S ratio (VSR), VFA/BMI, (VFA/H2) VFA/height2 (VFA/H2), VFA/weight, and VFA/WC were evaluated as well. Patients with VFA ≥ 100 cm^2^ were considered to have increased visceral adipose tissue (VFA (+)). The quartiles of VFA were set to further explore the association among VFA, left ventricular structure and function, and artery stiffness, respectively, in males and females.

### 2.5 Artery stiffness

Brachial‐ankle pulse wave velocity (baPWV) was applied to estimate the artery stiffness of patients with diabetes in this study.^22^ The baPWV was determined on the volume‐plethysmographic apparatus (BP‐203RPE II form PWV/ABI, Omron Healthcare Co. Ltd., Kyoto, Japan). The baPWV of both sides was measured, and their average value was calculated to carry out statistical analysis.

### 2.6 Echocardiographic examination and parameters

Echocardiographic examination was performed in all patients using a commercially available echocardiography system (Phillips EPIQ‐7c, Holland). Left atrial diameter (LAD), interventricular septal thickness (IVST), left ventricular end‐diastolic diameter (LVEDD), left ventricular posterior wall thickness (LVPWT), left ventricular end‐systolic diameter (LVESD), left ventricular ejection fraction (LVEF), and aortic root inside diameter (AORD) according to the guidelines of the American Society of Echocardiography.^23^ Left ventricular systolic function is evaluated by LVEF. Relative wall thickness (RWT) was calculated using the formula, RWT = (IVST + LVPWT)/LVEDD. LV mass = 0.8 × 1.04 × [(IVS + LVID + PWT)^3^ − LVID^3^] + 0.6 g. LV mass index (LVMI) was evaluated as LVM/(height,m)^2.7^, which offers accurate estimation of LV hypertrophy in the heart structure, particularly in obese subjects.^24^

### 2.7 Statistical analyses

Kolmogorov–Smirnov (KS) test was applied on all continuous data. Data were expressed in the tables and text as mean ± SD or median (25%, 75%) for continuous variables, and as frequency (percentages) for categorical variables. One‐way ANOVA and independent *t* test were used to make comparisons for normally distributed values, and Mann–Whitney test was used for non‐normally distributed values. Categorical variables were analyzed by the chi‐squared (χ^2^) test or Fisher exact test. The relationship between clinical variables and LV structure and function was assessed using Spearman correlation analysis. Linear regression modeling was used to assess associations of fat parameters and echocardiographic data (LV structure and function). To determine independent variables of LVEF, LVMI, and arterial stiffness, multiple linear regression was performed with variables adjusted for as detailed. A two‐sided *p* < 0.05 was considered to be statistically significant. Statistical Package for the Social Science (SPSS Version 25.0, IBM) was applied in this study.

## 3 RESULTS

### 3.1 Clinical data

**FIGURE 1:**
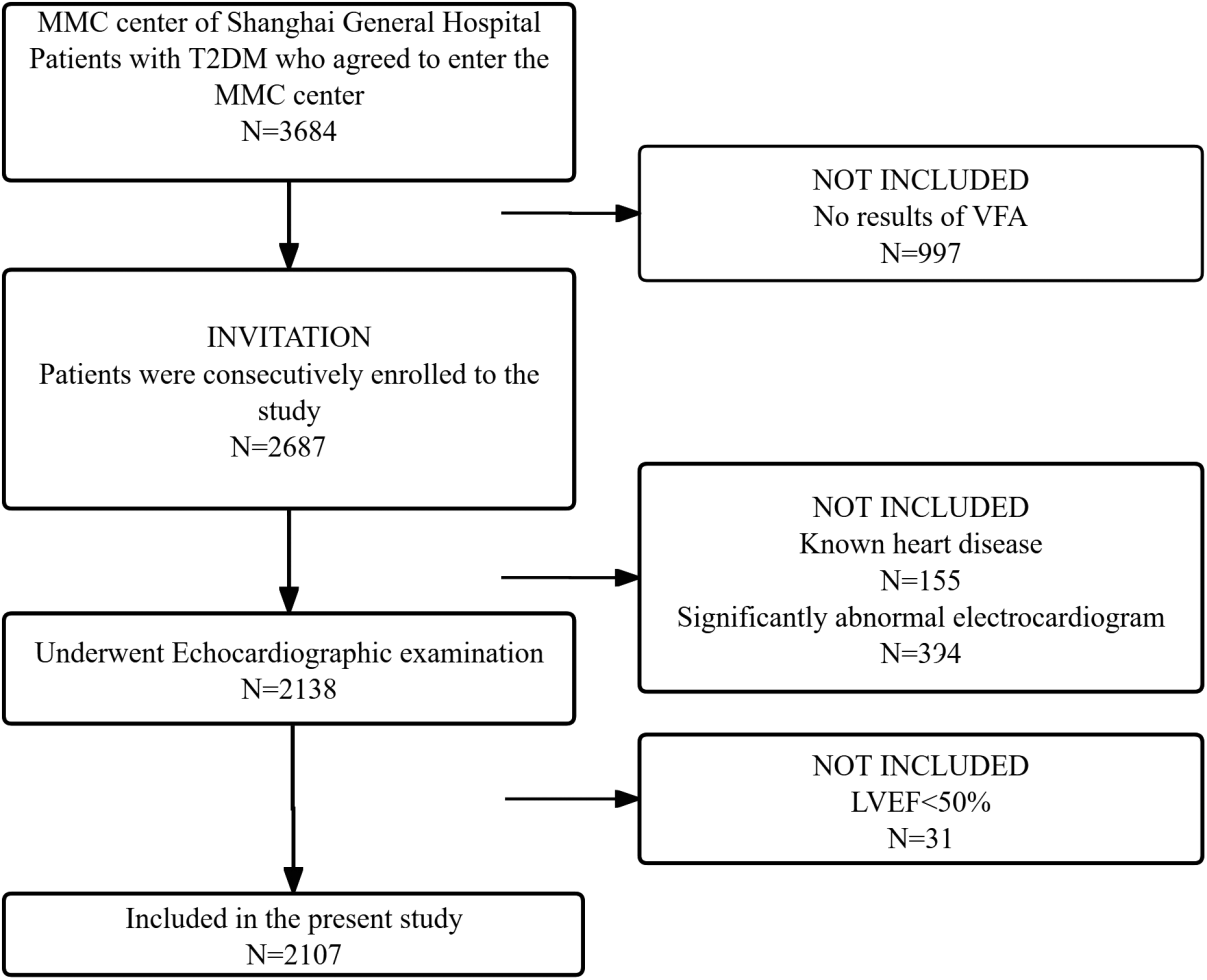
Flow diagram of the exclusion process of the study.

Among 2107 participants enrolled in the current study (Figure 1), the median age was 52 years, and 34.3% were female (Table 1). Mean BMI was 26.02 kg/m^2^ among males and 25.59 kg/m^2^ among females, and the mean VFA was 110.07 cm^2^ among males and 95.04 cm^2^ among females. The proportion of patients with VFA (+) was higher (58.7% vs. 39.3%) in males than in females. Men and women showed significant differences in most clinical variables including anthropometric measurements, age, duration of diabetes, the prevalence of hypertension and smoking history, fat deposal, baPWV, and ventricular structure and function (*p* < 0.05); LDL‐C, fasting C‐peptide, glucose, and insulin level were not significantly different according to sex (*p* > 0.05). Proportions of patients using glucosidase inhibitors, thiazolidinediones, insulin, CCBs, ACEI/ARBs, and antiplatelet medications in the female group were higher, and DPP‐4i and SGLT‐2i were lower than in male (*p* < 0.05). No statistical differences were found in the using of GLP‐1RA, sulfonylureas/nateglinide/repaglinide, biguanides, and lipid‐lowering medications between two groups.

**TABLE 1:** Clinical characteristics of the total participants according to sex.

Variable	Male (*n* = 1385)	Female (*n* = 722)	*t*/*z*/*χ* ^2^	*p* value
Age, years	49 (39, 58)	56 (45, 63)	−8.734	<0.001
Age of onset, years	45 (37, 53)	50 (41, 57)	−7.233	<0.001
Duration of DM, months	10 (0, 83)	30 (1122)	−5.506	<0.001
Hypertension history, *n* (%)	499 (36.0)	333 (46.1)	20.233	<0.001
Smoking history^a^, *n* (%)	716/162/507 (51.7/11.7/36.6)	701/8/13 (97.1/1.1/1.8)	444.337	<0.001
GLP‐1RA, *n* (%)	153 (11.0)	73 (10.7)	0.434	0.510
DPP‐4i, *n* (%)	723 (52.2)	338 (46.8)	5.511	0.019
Glucosidase inhibitors, *n* (%)	385 (27.8)	231 (32.0)	4.040	0.044
Thiazolidinediones, *n* (%)	194 (14.0)	151 (20.9)	16.535	<0.001
SGLT‐2i, *n* (%)	431 (29.8)	175 (24.2)	7.348	0.007
Sulfonylureas/nateglinide/repaglinide, *n* (%)	211 (15.2)	100 (13.9)	0.723	0.395
Biguanides, *n* (%)	1088 (78.6)	573 (79.4)	0.185	0.667
Insulin, *n* (%)	321 (23.2)	216 (29.9)	11.353	0.001
CCBs, *n* (%)	380 (27.4)	264 (36.6)	18.634	<0.001
ACEI/ARBs, *n* (%)	262 (18.9)	191 (26.5)	15.975	<0.001
Antiplatelet medications, *n* (%)	85 (6.1)	63 (8.7)	4.869	0.027
Lipid‐lowering medications, *n* (%)	696 (50.3)	373 (51.7)	0.377	0.539
DBP, mmHg	78.02 ± 10.84	75.04 ± 10.54	6.047	<0.001
SBP, mmHg	128.97 ± 17.15	130.22 ± 17.95	−1.559	0.119
BMI, kg/m^2^	26.02 ± 3.51	25.59 ± 3.83	2.532	0.011
WC, cm	92.80 ± 8.63	89.73 ± 9.70	7.428	<0.001
NC, cm	40.53 ± 3.24	36.21 ± 3.53	28.176	<0.001
WHR	0.95 ± 0.05	0.93 ± 0.07	5.902	<0.001
HOMA‐IR	3.08 (2.64, 3.79)	3.08 (2.60, 3.66)	−0.568	0.570
FPG, mmol/L	8.33 ± 2.78	8.32 ± 2.67	0.027	0.978
FIns, pmol/L	51.95 (34.60, 81.50)	53.60 (36.10, 84.40)	−0.649	0.516
FC‐P, pmol/L	573.18 ± 290.50	552.48 ± 265.96	1.597	0.111
HbA1c, %	8.55 ± 2.14	8.31 ± 2.02	2.550	0.011
GA, %	21.76 ± 7.12±	21.37 ± 7.03	1.166	0.244
TC, mmol/L	4.99 ± 1.22±	5.10 ± 1.21	−1.955	0.051
TG, mmol/L	1.70 (1.20, 2.53)	1.56 (1.15, 2.10)	−3.692	<0.001
HDL‐C, mmol/L	0.97 ± 0.24	1.13 ± 0.29	−12.557	<0.001
LDL‐C, mmol/L	2.82 ± 0.98	2.84 ± 0.95	−0.557	0.577
UA, mmol/L	360.27 ± 95.74	306.02 ± 92.90	12.398	<0.001
BUN, mmol/L	5.54 ± 1.66	5.12 ± 1.54	5.589	<0.001
Scr, μmol/L	66.57 ± 19.05	48.91 ± 13.57	22.022	<0.001
e‐GFR, ml/min/1.73 m^2^	137.34 ± 49.33	151.13 ± 62.13	−5.524	<0.001
Fat deposal				
SFA, cm^2^	184.81 ± 64.48	194.45 ± 72.55	−3.002	<0.001
VFA, cm^2^	110.07 ± 42.74	95.04 ± 33.59	8.220	<0.001
VSR	0.60 ± 0.17	0.51 ± 0.16	11.783	<0.001
VFA/BMI	4.14 ± 1.28	3.66 ± 0.98	9.704	<0.001
VFA/H^2^, cm^2^/m^2^	37.51 ± 14.50	37.92 ± 13.37	−0.631	0.528
VFA/weight, cm^2^/kg	1.41 ± 0.43	1.46 ± 0.40	−2.453	0.014
baPWV, cm/s	1515.83 ± 311.18	1600.52 ± 372.60	−5.510	<0.001
Ventricular structure and function				
LAD, mm	35.59 ± 3.62	34.65 ± 3.95	5.513	<0.001
IVST, mm	8.65 ± 1.16	8.31 ± 1.21	6.184	<0.001
LVEDD, mm	48.97 ± 3.60	47.16 ± 3.37	11.217	<0.001
LVPWT, mm	8.49 ± 0.86	8.22 ± 1.23	5.953	<0.001
LVESD, mm	31.87 ± 3.13	30.50 ± 2.88	9.813	<0.001
LVEF, %	63.97 ± 2.59	64.30 ± 2.49	−2.769	0.006
AORD, mm	33.53 ± 3.65	31.02 ± 2.91	17.124	<0.001
RWT	0.35 ± 0.07	0.35 ± 0.05	0.190	0.849
LV mass, g	144.9 ± 30.80	128.87 ± 24.90	12.901	<0.001
LVMI, g/m^2.7^	34.09 ± 7.81	37.40 ± 7.79	−9.244	<0.001

*Note*: Values are presented as number (%), mean ± standard deviation, or median (25%, 75%) as appropriate. Student's *t* test for comparison of normally distributed quantitative variables between two groups. χ^2^ test for comparison of qualitative variables between two groups.

Abbreviations: ACEI/ARBs, angiotensin‐converting enzyme inhibitors/angiotensin II receptor blocker; AORD, aortic root inside diameter; baPWV, brachial‐ankle pulse wave velocity; BMI, body mass index; BUN, blood urea nitrogen; CCBs, calcium channel blockers; DBP, diastolic blood pressure; DM, diabetes mellitus; DPP‐4i, dipeptidyl peptidase‐4 inhibitor; e‐GFR, estimated glomerular filtration rate; FC‐P, fasting C‐peptide; FIns, fasting insulin; FPG, fasting plama glucose; GA, glycated albumin; GLP‐1RA, glucagon‐like peptide‐1 receptor agonist; HbA1c, glycosylated hemoglobin; HDL‐C, high‐density cholesterol; HOMA‐IR, homeostasis model assessment of insulin resistance; IVST, interventricular septal thickness; LAD, left atrial diameter; LDL‐C, low‐density cholesterol; LV mass, left ventricular mass; LVEDD, left ventricular end‐diastolic diameter; LVEF, left ventricular ejection fraction; LVESD, left ventricular end‐systolic diameter; LVMI, left ventricular mass index; LVPWT, left ventricular posterior wall thickness; NC, neck circumference; RWT, relative wall thickness; SBP, systolic blood pressure; Scr, serum creatinine; SFA, subcutaneous fat area; SGLT‐2i, sodium‐glucose cotransporter‐2 inhibitor; TC, total cholesterol; TG, triglycerides; UA, uric acid; VFA, visceral fat area; VSR, visceral‐to‐subcutaneous fat ratio; WC, waist circumference; WHR, waist‐to‐hip ratio.

^a^ The 0/1/2 represents non‐smoking/former smoking/current smoking.

As shown in Table 2, both males and females in the highest VFA quartile (Q4) have higher proportion of hypertension; increased DBP and SBP, HOMA‐IR, Fins, FC‐P, TC, TG, fat deposal parameters, LAD, IVST, LVEDD, LVPWT, LVESD, AORT, LV mass, and LVMI; and lower HDL‐C than those in Q1 (*p* < 0.05). Nevertheless, values of LVEF were decreased, and baPWV was increased in the Q4 than in the Q1 in females (*p* < 0.05) but not in males. Proportions of patients using GLP‐1RA, DPP‐4i, and CCBs increased, and thiazolidinediones decreased with higher quartiles of VFA in both male and female groups (*p*_trend_ < 0.05).

**TABLE 2:** Clinical characteristics of the total participants according to the quartiles of VFA in male and female.

Variable	Male						Female					
	Quartile 1	Quartile 2	Quartile 3	Quartile 4	*F*/*χ* ^2^	*p*_*t*rend_	Quartile 1	Quartile 2	Quartile 3	Quartile 4	*F*/*χ* ^2^	*p*_*t*rend_
VEF	(5.0–83.6)	(83.8–107.7)	(107.8–133.8)	(133.9–334.0)			(19.5–72.2)	(72.3–90.9)	(91.0–112.8)	(112.9–223.8)		
	*N* = 346	*N* = 346	*N* = 346	*N* = 347			*N* = 181	*N* = 180	*N* = 180	*N* = 181		
Age, years	53 (43, 61)	48 (40, 57)	47 (38, 56)	49 (38, 57)	15.652	0.001	55 (44, 61)	55 (49, 63)	56 (44, 65)	57 (44, 65)	5.743	0.125
Age of onset, years	46 (38, 55)	45 (38, 52)	44 (36, 52)	44 (37, 54)	2.918	0.404	49 (41, 55)	51 (42, 56)	50 (43, 59)	50 (40, 58)	1.661	0.198
Duration of DM, months	36 (1122)	3 (0,61)	7 (0, 73)	7 (0, 74)	27.490	<0.001	44 (1122)	26 (1122)	23 (1, 96)	37 (1122)	1.790	0.617
Hypertension history, *n* (%)	91 (26.3)	122 (32.4)	128 (37.0)	168 (48.4)	39.455	<0.001	5 (31.7)	79 (43.6)	87 (48.3)	110 (60.8)	31.572	<0.001
^※^Smoking history, *n* (%)	183/30/133	174/49/123	187/37/122	172/46/129	7.053	0.316	174/1/5	177/2/2	175/2/3	175/3/3	2.484	0.870
GLP‐1RA, *n* (%)	1 (0.3)	6 (1.7)	38 (11.0)	108 (31.1)	213.628	<0.001	1 (0.6)	3 (1.7)	20 (11.1)	49 (27.1)	89.806	<0.001
DPP‐4i, *n* (%)	74 (21.4)	186 (53.8)	239 (69.1)	224 (64.6)	192.703	<0.001	36 (19.9)	81 (45.0)	110 (61.1)	111 (60.2)	89.023	<0.001
Glucosidase inhibitors, *n* (%)	94 (27.2)	51 (14.7)	71 (20.5)	169 (48.7)	114.152	<0.001	62 (34.3)	34 (18.9)	47 (26.1)	88 (48.6)	40.488	<0.001
Thiazolidinediones, *n* (%)	151 (43.6)	28 (8.1)	14 (4.0)	1 (0.3)	345.034	<0.001	99 (54.7)	39 (21.7)	8 (4.4)	5 (2.8)	190.522	<0.001
SGLT‐2i, *n* (%)	36 (10.4)	62 (17.9)	118 (34.1)	197 (56.8)	209.224	<0.001	34 (18.8)	50 (27.8)	58 (32.2)	33 (18.2)	0.038	0.846
Sulfonylureas/nateglinide/repaglinide, *n* (%)	47 (13.6)	61 (17.6)	66 (19.1)	37 (10.7)	11.836	0.008	24 (13.3)	26 (14.4)	33 (18.3)	17 (9.4)	6.153	0.104
Biguanides (%)	272 (78.6)	298 (86.1)	273 (78.9)	245 (70.6)	24.821	<0.001	111 (61.3)	157 (87.2)	160 (88.9)	145 (80.1)	52.777	<0.001
Insulin, *n* (%)	160 (46.2)	56 (16.2)	40 (11.6)	65 (18.7)	142.96	<0.001	101 (55.8)	44 (24.4)	25 (13.9)	46 (25.4)	84.215	<0.001
CCBs, *n* (%)	72 (18.9)	94 (24.7)	99 (26.1)	115 (30.3)	13.558	0.004	41 (22.7)	65 (36.1)	70 (38.9)	88 (48.6)	26.878	<0.001
ACEI/ARBs, *n* (%)	50 (14.5)	64 (18.5)	70 (20.2)	78 (22.5)	7.798	0.05	41 (22.7)	49 (27.2)	50 (27.8)	51 (28.2)	1.838	0.607
Antiplatelet medications, *n* (%)	27 (7.8)	19 (5.5)	23 (6.6)	16 (4.6)	3.478	0.324	21 (11.6)	14 (7.8)	18 (10.0)	10 (5.5)	4.779	0.189
Lipid‐lowering medications, *n* (%)	194 (56.1)	170 (49.1)	164 (47.4)	168 (48.4)	6.452	0.092	116 (64.1)	80 (44.4)	89 (49.4)	88 (48.6)	15.973	0.001
DBP, mmHg	73.51 ± 10.17	78.06 ± 9.95	79.16 ± 10.28	81.35 ± 11.41	95.715	<0.001	73.03 ± 9.66	74.97 ± 10.80	75.29 ± 10.71	76.86 ± 10.70	11.441	0.001
SBP, mmHg	122.91 ± 16.64	128.3 ± 16.19	130.08 ± 15.71	134.58 ± 17.98	84.533	<0.001	125.68 ± 17.97	131.59 ± 19.22	129.86 ± 16.50	133.73 ± 17.14	14.438	<0.001
BMI, kg/m^2^	22.91 ± 2.21	25.12 ± 2.04	26.52 ± 2.25	29.5 ± 3.5	1177.614	<0.001	22.52 ± 2.29	24.47 ± 2.34	26.13 ± 2.60	29.25 ± 4.13	498.843	<0.001
WC, cm	85.67 ± 6.38	90.27 ± 5.03	94.07 ± 5.77	101.17 ± 8.36	1036.797	<0.001	82.19 ± 7.13	87.25 ± 6.62	90.80 ± 6.67	98.64 ± 9.70	432.627	<0.001
NC, cm	38.51 ± 2.84	40.01 ± 2.75	41.07 ± 2.82	42.54 ± 3.15	357.204	<0.001	34.29 ± 2.72	35.93 ± 3.30	36.73 ± 3.46	37.88 ± 3.59	111.624	<0.001
WHR	0.92 ± 0.05	0.94 ± 0.04	0.950.04	0.98 ± 0.05	315.606	<0.001	0.9 ± 0.07	0.920.06	0.94 ± 0.08	0.97 ± 0.06	83.456	<0.001
HOMA‐IR	2.54 (2.22, 3.05)	2.98 (2.59, 3.51)	3.16 (2.81, 3.82)	3.66 (3.06, 4.44)	197.776	<0.001	2.69 (2.29, 3.27)	3.02 (2.59, 3.47)	3.14 (2.73, 3.76)	3.73 (3.04, 4.40)	65.832	<0.001
FPG, mmol/L	8.02 ± 2.81	8.23 ± 2.69	8.35 ± 2.85	8.7 ± 2.74	10.315	0.001	8.18 ± 3.04±	8.29 ± 2.56	8.11 ± 2.30	8.7 ± 2.72	2.471	0.116
FIns, pmol/L	33.70 (23.45, 51.50)	46.50 (32.70, 63.60)	59.00 (42.10, 83.10)	71.80 (50.20109.05)	145.040	<0.001	39.20 (27.40, 60.40)	48.00 (33.05, 66.40)	58.50 (39.8 66.40)	74.60 (50.50103.85)	52.696	0.084
FC‐P, pmol/L	390.39 ± 221.19	524.16 ± 236.05	604.22 ± 245.08	773.36 ± 310.41	400.723	<0.001	406.18 ± 189.78	509.62 ± 211.99	589.92 ± 256.77	703.6 ± 298.98	144.456	<0.001
HbA1c, %	8.81 ± 2.48	8.49 ± 2.08	8.45 ± 2.09	8.46 ± 1.88	4.660	0.031	8.21 ± 2.23	8.23 ± 1.98	8.24 ± 1.93	8.54 ± 1.91	2.316	0.128
GA, %	24.16 ± 8.28	21.52 ± 6.67	20.95 ± 6.5	20.38 ± 6.27	50.101	<0.001	22.73 ± 9.11	21.48 ± 6.52	20.62 ± 5.88	20.66 ± 5.92	9.079	0.003
TC, mmol/L	4.8 ± 1.14	4.93 ± 1.18	5.12 ± 1.3	5.09 ± 1.21	13.177	<0.001	4.79 ± 1.15	5.2 ± 1.18	5.2 ± 1.14	5.19 ± 1.33	8.693	0.003
TG, mmol/L	1.18 (0.88, 1.81)	1.63 (1.18, 2.41)	1.89 (1.43, 2.87)	2.11 (1.45, 3.09)	98.985	<0.001	1.22 (0.95, 1.61)	1.58 (1.16, 2.23)	1.69 (1.30, 2.32)	1.78 (1.36, 2.55)	50.556	<0.001
HDL‐C, mmol/L	1.09 ± 0.31	0.96 ± 0.2	0.93 ± 0.19	0.9 ± 0.2	104.465	<0.001	1.24 ± 0.33	1.12 ± 0.28	1.12 ± 0.27	1.04 ± 0.23	37.907	<0.001
LDL‐C, mmol/L	2.67 ± 0.95	2.81 ± 0.98	2.92 ± 1.00	2.88 ± 0.98	9.830	0.002	2.95 ± 0.91	2.93 ± 0.99	2.90 ± 0.92	2.84 ± 0.95	8.606	0.003
UA, mmol/L	328.07 ± 99.97	360.02 ± 89.47	366.72 ± 90.69	386.44 ± 93.45	64.619	<0.001	271.22 ± 77.34	312.74 ± 106.17	305.84 ± 82.64	334.2 ± 92.01	36.375	<0.001
BUN, mmol/L	5.54 ± 1.69	5.63 ± 1.63	5.41 ± 1.59	5.57 ± 1.71	0.085	0.770	4.99 ± 1.41	5.13 ± 1.48	5.13 ± 1.47	5.23 ± 1.76	1.965	0.161
Scr, μmol/L	65.66 ± 18.55	66.67 ± 21.17	66.07 ± 14.52	67.87 ± 21.23	1.716	0.190	48.39 ± 13.28	49.36 ± 11.63	48.67 ± 12.62	49.23 ± 16.38	0.164	0.686
e‐GFR, mL/min/1.73 m^2^	116.94 ± 41.69	130.47 ± 35.91	143.59 ± 47.7	158.4 ± 59.14	146.710	<0.001	131.82 ± 43.67	139.56 ± 50.60	155.92 ± 61.55	177.43 ± 77.69	58.828	<0.001
Fat deposal												
SFA, cm^2^	127.46 ± 31.18	168.98 ± 40.07	194.72 ± 42.19	247.91 ± 68.21	1151.218	<0.001	143.81 ± 45.64	171.84 ± 47.08	207.16 ± 63.38	254.76 ± 76.83	344.012	<0.001
VSR	0.47 ± 0.15	0.6 ± 0.14	0.65 ± 0.15	0.7 ± 0.17	423.743	<0.001	0.42 ± 0.13	0.51 ± 0.14	0.54 ± 0.18	0.58 ± 0.14	109.428	<0.001
VFA/BMI	2.57 ± 0.82	3.84 ± 0.37	4.56 ± 0.45	5.6 ± 0.8	4039.754	<0.001	2.51 ± 0.56	3.38 ± 0.36	3.92 ± 0.39	4.81 ± 0.68	1887.882	<0.001
VFA/H^2^, cm^2^/m^2^	20.63 ± 6.85	32.92 ± 3.42	40.71 ± 4.01	55.75 ± 10.77	4650.523	<0.001	22.74 ± 5.34	32.96 ± 3.17	40.47 ± 3.98	55.46 ± 9.52	1887.882	<0.001
VFA/weight, cm^2^/kg	0.9 ± 0.29	1.32 ± 0.15	1.54 ± 0.19	1.89 ± 0.3	3156.184	<0.001	1.01 ± 0.23	1.36 ± 0.17	1.56 ± 0.19	1.91 ± 0.31	1431.874	<0.001
baPWV, cm/s	1517.62 ± 346.13	1515.00 ± 302.95	1495.50 ± 283.88	1535.30 ± 308.63	0.199	0.656	1550.12 ± 364.56	1630.45 ± 368.45	1551.06 ± 360.22	1670.28 ± 385.31	5.174	0.023
Ventricular structure												
LAD, mm	34.51 ± 3.77	35.15 ± 2.9	35.79 ± 2.95	36.9 ± 4.25	85.319	<0.001	32.85 ± 3.13	34.33 ± 3.67	35.1 ± 3.52	36.29 ± 4.54	78.921	<0.001
IVST, mm	8.49 ± 1.2	8.48 ± 0.9	8.66 ± 1.18	8.95 ± 1.26	32.812	<0.001	8.03 ± 0.67	8.32 ± 0.92	8.25 ± 0.69	8.62 ± 1.97	18.460	<0.001
LVEDD, mm	48.43 ± 3.29	48.56 ± 4.11	49.2 ± 3.19	49.69 ± 3.6	26.387	<0.001	8.03 ± 3.31	8.32 ± 3.58	8.25 ± 3.14	8.62 ± 3.07	40.771	<0.001
LVPWT, mm	8.34 ± 0.84	8.37 ± 0.8	8.5 ± 0.83	8.77 ± 0.91	48.385	<0.001	7.97 ± 0.64	8.35 ± 1.95	8.13 ± 0.63	8.43 ± 1.15	8.297	0.004
LVESD, mm	31.62 ± 2.93	31.56 ± 3.20	32.01 ± 3.20	32.30 ± 3.16	11.202	0.001	29.72 ± 2.37	30.45 ± 3.39	30.78 ± 2.46	31.04 ± 3.04	20.678	<0.001
LVEF, %	63.95 ± 2.64	64.14 ± 2.60	64.01 ± 2.62	63.78 ± 2.52	1.034	0.309	64.6 ± 2.39	64.17 ± 2.51	64.43 ± 2.38	63.99 ± 2.65	3.610	0.058
AORD, mm	32.79 ± 3.60	33.61 ± 3.33	33.30 ± 3.58	34.41 ± 3.89	27.486	<0.001	30.53 ± 2.99	30.93 ± 2.88	30.94 ± 2.65	31.68 ± 3.01	13.041	<0.001
RWT	0.35 ± 0.04	0.35 ± 0.11	0.35 ± 0.04	0.36 ± 0.04	2.178	0.140	0.35 ± 0.04	0.36 ± 0.06	0.35 ± 0.04	0.35 ± 0.05	0.205	0.651
LV mass, g	138.64 ± 28.48	139.81 ± 28.69	145.72 ± 27.18	155.42 ± 35.38	60.413	<0.001	119.42 ± 20.75	129.72 ± 37.62	129.27 ± 19.89	140.44 ± 37.57	38.680	<0.001
LVMI, g/m^2.7^	33.6 ± 7.23	33.04 ± 7.82	33.71 ± 6.81	36.02 ± 8.92	18.129	<0.001	35.15 ± 7.30	37.69 ± 9.89	37.36 ± 6.75	40.36 ± 11.93	24.976	<0.001

Abbreviations: ACEI/ARBs, angiotensin‐converting enzyme inhibitors/angiotensin II receptor blocker; AORD, aortic root inside diameter; baPWV, brachial‐ankle pulse wave velocity; BMI, body mass index; BUN, blood urea nitrogen; CCBs, calcium channel blockers; DBP, diastolic blood pressure; DM, diabetes mellitus; DPP‐4i, dipeptidyl peptidase‐4 inhibitor; e‐GFR, estimated glomerular filtration rate; FC‐P, fasting C‐peptide; FIns, fasting insulin; FPG, fasting plama glucose; GA, glycated albumin; GLP‐1RA, glucagon‐like peptide‐1 receptor agonist; HbA1c, glycosylated hemoglobin; HDL‐C, high‐density cholesterol; HOMA‐IR, homeostasis model assessment of insulin resistance; IVST, interventricular septal thickness; LAD, left atrial diameter; LDL‐C, low‐density cholesterol; LV mass, left ventricular mass; LVEDD, left ventricular end‐diastolic diameter; LVEF, left ventricular ejection fraction; LVESD, left ventricular end‐systolic diameter; LVMI, left ventricular mass index; LVPWT, left ventricular posterior wall thickness; NC, neck circumference; RWT, relative wall thickness; SBP, systolic blood pressure; Scr, serum creatinine; SFA, subcutaneous fat area; SGLT‐2i, sodium‐glucose cotransporter‐2 inhibitor; TC, total cholesterol; TG, triglycerides; UA, uric acid; VFA, visceral fat area; VSR, visceral‐to‐subcutaneous fat ratio; WC, waist circumference; WHR, waist‐to‐hip ratio.

※ The 0/1/2 represents non‐smoking/former smoking/current smoking.

### 3.2 Univariable association of clinical variables with visceral fat area, cardiac parameters, and baPWV

**FIGURE 2:**
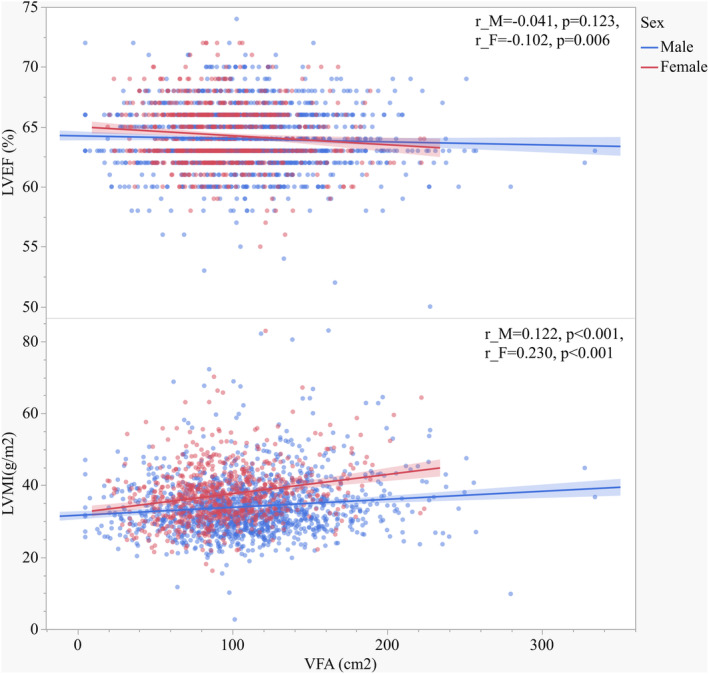
Scatter plot and Spearman correlation coefficient of LVEF and LVMI according to VFA in different sexes. VFA, visceral fat area; LVEF, left ventricular ejection fraction; LVMI, left ventricular mass index; r_M, Spearman rank correlation coefficients of male; r_F, Spearman rank correlation coefficients of female.

Tables 3 and 4 show the association of clinical variables with cardiac parameters and baPWV in males (Table 3) and females (Table 4). Values of VFA and SFA were positively associated with LAD, IVST, LVEDD, LVPWT, LVESD, AORD, LV mass, and LVMI (Figure 2) in both males and females. LVEF was negatively associated with age, hypertension history, using of GLP‐1RA, CCBs, lipid‐lowering medications, BMI, WC, WHR, FPG, FC‐P, HbA1c, GA, HOMA‐IR, Cr, and baPWV in males, but negatively associated with age, VFA (Figure 2), VSR, VFA/BMI, VFA/H^2^, VFA/weight, and BUN in females. BaPWV was positively associated with duration of diabetes, age, hypertension history, DBP, SBP, using of CCBs and ACEI/ARBs, BMI, VSR, VFA/H^2^, VFA/weight, FPG, LAD, IVST, AORD, LV mass, and LVMI and negatively associated with e‐GFR in both males and females. Remarkably, baPWV was positively associated with using of lipid‐lowering medications, WHR, LVPWT, and RWT and negatively associated with LVEF in males. LVMI was positively associated with diabetes duration, age, hypertension history, SBP, using of DDP‐4i, CCBs, WC, WHR, VFA, SFA, VFA/H^2^, VFA/weight, BUN, Cr, and baPWV, but negatively associated with using of thiazolidinediones, insulin, and e‐GFR in both males and females. For sex stratification analysis, LVMI was positively associated with using of GLP‐1RA, glucosidase inhibitors, SGLT‐2i, ACEI/ARBs, antiplatelet medications, lipid‐lowering medications, FC‐P and HOMA‐IR in males, and respectively, positively associated with using of biguanides and VFA/BMI in females.

**TABLE 3:** Correlation of clinical variables with cardiac parameters and baPWV in male patients.

		LAD	IVST	LVEDD	LVPWT	LVESD	AORD	LVEF	RWT	LV mass	LVMI	baPWV
Duration	*r*	0.070**	0.079**	0.003	0.087**	0.007	0.072**	−0.046	0.037	0.057*	0.087**	0.337**
	*p*	0.009	0.003	0.914	0.001	0.804	0.007	0.090	0.173	0.035	0.001	<0.001
Age	*r*	0.223**	0.189**	0.092**	0.207**	0.123**	0.171**	−0.096**	0.109**	0.204**	0.233**	0.272**
	*p*	<0.001	<0.001	0.001	<0.001	<0.001	<0.001	<0.001	<0.001	<0.001	<0.001	<0.001
Hypertension history	*r*	0.166**	0.112**	0.013	0.147**	0.030	0.208**	−0.089**	0.086**	0.100**	0.227**	0.502**
	*p*	<0.001	<0.001	0.635	<0.001	0.264	<0.001	0.001	0.001	<0.001	<0.001	<0.001
DBP	*r*	0.106**	0.113**	0.060*	0.139**	0.065*	0.080**	−0.011	0.064*	0.129**	0.087**	0.243**
	*p*	<0.001	<0.001	0.026	<0.001	0.016	0.003	0.686	0.016	<0.001	0.001	<0.001
SBP	*r*	0.193**	0.189**	0.116**	0.226**	0.093**	0.087**	−0.021	0.082**	0.223**	0.212**	0.418**
	*p*	<0.001	<0.001	<0.001	<0.001	0.001	0.001	0.428	0.002	<0.001	<0.001	<0.001
GLP‐1RA	*r*	0.177**	0.136**	0.127**	0.133**	0.132**	0.094**	−0.062*	0.043	0.168**	0.113**	−0.047
	*p*	<0.001	<0.001	<0.001	<0.001	<0.001	<0.001	0.021	0.109	<0.001	<0.001	0.079
DPP‐4i	*r*	0.167**	0.070**	0.140**	0.079**	0.085**	0.051	−0.007	−0.024	0.129**	0.122**	−0.01
	*p*	<0.001	0.009	<0.001	0.003	0.002	0.060	0.782	0.363	<0.001	<0.001	0.701
Glucosidase inhibitors	*r*	0.078**	0.071**	0.105**	0.083**	0.075**	0.048	−0.008	−0.007	0.107**	0.081**	−0.066*
	*p*	0.004	0.008	<0.001	0.002	0.005	0.073	0.759	0.797	<0.001	0.003	0.015
Thiazolidinediones	*r*	−0.262**	−0.168**	−0.193**	−0.174**	−0.178**	−0.122**	0.045	−0.025	−0.230**	−0.184**	−0.012
	*p*	<0.001	<0.001	<0.001	<0.001	<0.001	<0.001	0.098	0.347	<0.001	<0.001	0.664
SGLT‐2i	*r*	0.182**	0.136**	0.107**	0.133**	0.107**	0.080**	−0.044	0.058*	0.149**	0.120**	0.005
	*p*	<0.001	<0.001	<0.001	<0.001	<0.001	0.003	0.100	0.031	<0.001	<0.001	0.858
Sulfonylureas/nateglinide/repaglinide	*r*	−0.035	−0.022	0.001	−0.011	−0.015	−0.015	−0.019	−0.025	−0.010	0.004	−0.029
	*p*	0.192	0.422	0.981	0.681	0.589	0.567	0.484	0.346	0.702	0.89	0.275
Biguanides	*r*	0.017	−0.045	0.045	−0.042	0.016	0.023	0.020	−0.066*	0.002	0.007	−0.002
	*p*	0.515	0.098	0.097	0.118	0.564	0.401	0.466	0.014	0.938	0.803	0.952
Insulin	*r*	−0.188**	−0.089**	−0.111**	−0.094**	−0.086**	−0.081**	−0.010	−0.016	−0.126**	−0.132**	−0.036
	*p*	<0.001	0.001	<0.001	<0.001	0.001	0.003	0.713	0.562	<0.001	<0.001	0.185
CCBs	*r*	0.190**	0.232**	0.101**	0.235**	0.095**	0.144**	−0.063*	0.150**	0.224**	0.272**	0.251**
	*p*	<0.001	<0.001	<0.001	<0.001	<0.001	<0.001	0.019	<0.001	<0.001	<0.001	<0.001
ACEI/ARBs	*r*	0.032	−0.088**	−0.058*	−0.079**	−0.015	0.054*	−0.011	−0.05	−0.099**	−0.094**	0.146**
	*p*	0.230	0.001	0.032	0.003	0.566	0.045	0.679	0.064	<0.001	<0.001	<0.001
Antiplatelet medications	*r*	0.035	0.024	−0.009	0.022	0.001	0.038	−0.007	0.017	0.021	0.064*	0.027
	*p*	0.199	0.376	0.749	0.413	0.974	0.155	0.792	0.526	0.446	0.018	0.318
Lipid‐lowering medications	*r*	0.057*	−0.002	0.029	−0.001	0.046	0.05	−0.074**	−0.030	0.023	0.076**	0.111**
	*p*	0.035	0.928	0.288	0.966	0.084	0.065	0.006	0.258	0.398	0.005	<0.001
BMI	*r*	0.326**	0.187**	0.273**	0.229**	0.222**	0.146**	−0.074**	0.014	0.318**	0.252**	−0.059*
	*p*	<0.001	<0.001	<0.001	<0.001	<0.001	<0.001	0.006	0.596	<0.001	<0.001	0.029
NC	*r*	0.230**	0.128**	0.194**	0.155**	0.143**	0.136**	−0.009	0.013	0.219**	0.109**	−0.020
	*p*	<0.001	<0.001	<0.001	<0.001	<0.001	<0.001	0.725	0.622	<0.001	<0.001	0.470
WC	*r*	0.331**	0.183**	0.241**	0.227**	0.212**	0.186**	−0.093**	0.015	0.295**	0.146**	−0.009
	*p*	<0.001	<0.001	<0.001	<0.001	<0.001	<0.001	0.001	0.579	<0.001	<0.001	0.736
WHR	*r*	0.231**	0.110**	0.118**	0.135**	0.121**	0.126**	−0.088**	0.008	0.158**	0.104**	0.089**
	*p*	<0.001	<0.001	<0.001	<0.001	<0.001	<0.001	0.001	0.779	<0.001	<0.001	0.001
VFA	*r*	0.251**	0.148**	0.149**	0.190**	0.125**	0.150**	−0.041	0.039	0.218**	0.122**	0.025
	*p*	<0.001	<0.001	<0.001	<0.001	<0.001	<0.001	0.123	0.146	<0.001	<0.001	0.359
SFA	*r*	0.296**	0.147**	0.231**	0.186**	0.180**	0.158**	−0.041	0.002	0.260**	0.102**	−0.060
	*p*	<0.001	<0.001	<0.001	<0.001	<0.001	<0.001	0.125	0.955	<0.001	<0.001	0.027
VSR	*r*	−0.024	0.020	−0.073**	0.026	−0.053*	0.014	0.001	0.049	−0.020	0.028	0.090**
	*p*	0.374	0.465	0.007	0.338	0.049	0.605	0.962	0.069	0.449	0.299	0.001
VFA/BMI	*r*	0.178**	0.103**	0.071**	0.137**	0.056*	0.127**	−0.011	0.043	0.133**	0.037	0.059*
	*p*	<0.001	<0.001	0.008	<0.001	0.036	<0.001	0.677	0.111	<0.001	0.166	0.029
VFA/H^2^	*r*	0.226**	0.145**	0.121**	0.180**	0.101**	0.126**	−0.041	0.049	0.198**	0.196**	0.071**
	*p*	<0.001	<0.001	<0.001	<0.001	<0.001	<0.001	0.130	0.066	<0.001	<0.001	0.009
VFA/weight	*r*	0.147**	0.099**	0.036	0.126**	0.028	0.095**	−0.011	0.056*	0.109**	0.129**	0.115**
	*p*	<0.001	<0.001	0.182	<0.001	0.295	<0.001	0.674	0.037	<0.001	<0.001	<0.001
FPG	*r*	0.013	0.027	−0.023	0.012	0.025	−0.043	−0.068*	0.048	0.005	−0.017	0.054*
	*p*	0.633	0.310	0.385	0.650	0.344	0.114	0.011	0.075	0.842	0.536	0.046
FIns	*r*	−0.009	−0.006	−0.014	−0.003	−0.003	0.019	−0.053	−0.006	−0.014	−0.030	−0.046
	*p*	0.755	0.830	0.644	0.912	0.912	0.539	0.081	0.849	0.634	0.323	0.127
FC‐P	*r*	0.178**	0.170**	0.106**	0.198**	0.098**	0.067*	−0.087**	0.084**	0.197**	0.112**	0.016
	*p*	<0.001	<0.001	<0.001	<0.001	<0.001	0.013	0.001	0.002	<0.001	<0.001	0.542
HbA1c	*r*	−0.074**	−0.029	−0.023	−0.042	0.011	−0.050	−0.070**	−0.007	−0.033	−0.036	−0.102**
	*p*	0.006	0.275	0.389	0.116	0.688	0.065	0.009	0.785	0.223	0.178	<0.001
GA	*r*	−0.059*	−0.049	−0.023	−0.068*	0.014	−0.020	−0.070**	−0.015	−0.050	−0.033	−0.051
	*p*	0.028	0.069	0.404	0.012	0.598	0.453	0.010	0.567	0.064	0.220	0.059
HOMA‐IR	*r*	0.136**	0.130**	0.055*	0.150**	0.079**	0.029	−0.098**	0.092**	0.137**	0.064*	0.051
	*p*	<0.001	<0.001	0.039	<0.001	0.003	0.284	<0.001	0.001	<0.001	0.017	0.059
TC	*r*	−0.054*	0.019	−0.039	0.017	−0.028	−0.052	−0.008	0.023	−0.006	−0.030	−0.073**
	*p*	0.045	0.490	0.150	0.526	0.310	0.055	0.775	0.400	0.828	0.266	0.007
TG	*r*	0.027	<0.001	0.014	0.011	0.041	−0.019	−0.001	0.007	0.011	−0.021	−0.025
	*p*	0.317	0.995	0.600	0.694	0.134	0.492	0.958	0.807	0.683	0.444	0.362
HDL‐c	*r*	−0.081**	−0.011	−0.070**	−0.020	−0.057*	−0.022	0.005	0.008	−0.050	−0.009	0.081**
	*p*	0.003	0.675	0.010	0.471	0.035	0.420	0.854	0.782	0.064	0.730	0.003
LDL‐c	*r*	−0.068*	0.011	−0.020	−0.006	−0.049	−0.060*	0.030	−0.013	−0.007	−0.030	−0.109**
	*p*	0.013	0.687	0.453	0.813	0.070	0.027	0.272	0.632	0.803	0.264	<0.001
UA	*r*	0.103**	0.048	0.020	0.061*	0.035	0.021	−0.035	0.041	0.056*	0.009	0.006
	*p*	<0.001	0.076	0.469	0.023	0.201	0.433	0.200	0.129	0.038	0.729	0.825
BUN	*r*	0.042	0.101**	−0.063*	0.125**	−0.057*	0.062*	−0.044	0.105**	0.043	0.084**	0.223
	*p*	0.117	<0.001	0.020	<0.001	0.035	0.021	0.103	<0.001	0.109	0.002	0
Cr	*r*	0.065*	0.111**	−0.026	0.145**	−0.008	0.043	−0.061*	0.107**	0.084**	0.094**	0.17
	*p*	0.017	<0.001	0.332	<0.001	0.769	0.109	0.025	<0.001	0.002	<0.001	<0.001
e‐GFR	*r*	0.084**	−0.004	0.190**	−0.017	0.144**	−0.021	0.002	−0.100**	0.105**	−0.074**	−0.351**
	*p*	0.002	0.872	<0.001	0.523	<0.001	0.449	0.939	<0.001	<0.001	0.006	<0.001
baPWV	r	0.133**	0.178**	0.021	0.216**	0.049	0.13**	−0.091**	0.116**	0.166**	0.257**	/
	p	<0.001	<0.001	0.438	<0.001	0.069	<0.001	0.001	<0.001	<0.001	<0.001	/

Abbreviations: ACEI/ARBs, angiotensin‐converting enzyme inhibitors/angiotensin II receptor blocker; AORD, aortic root inside diameter; baPWV, brachial‐ankle pulse wave velocity; BMI, body mass index; BUN, blood urea nitrogen; CCBs, calcium channel blockers; DBP, diastolic blood pressure; DM, diabetes mellitus; DPP‐4i, dipeptidyl peptidase‐4 inhibitor; e‐GFR, estimated glomerular filtration rate; FC‐P, fasting C‐peptide; FIns, fasting insulin; FPG, fasting plama glucose; GA, glycated albumin; GLP‐1RA, glucagon‐like peptide‐1 receptor agonist; HbA1c, glycosylated hemoglobin; HDL‐C, high‐density cholesterol; HOMA‐IR, homeostasis model assessment of insulin resistance; IVST, interventricular septal thickness; LAD, left atrial diameter; LDL‐C, low‐density cholesterol; LV mass, left ventricular mass; LVEDD, left ventricular end‐diastolic diameter; LVEF, left ventricular ejection fraction; LVESD, left ventricular end‐systolic diameter; LVMI, left ventricular mass index; LVPWT, left ventricular posterior wall thickness; NC, neck circumference; RWT, relative wall thickness; SBP, systolic blood pressure; Scr, serum creatinine; SFA, subcutaneous fat area; SGLT‐2i, sodium‐glucose cotransporter‐2 inhibitor; TC, total cholesterol; TG, triglycerides; UA, uric acid; VFA, visceral fat area; VSR, visceral‐to‐subcutaneous fat ratio; WC, waist circumference; WHR, waist‐to‐hip ratio.

* *p* < 0.05;

** *p* < 0.01.

**TABLE 4:** Correlation of clinical variables with cardiac parameters and baPWV in female patients.

		LAD	IVST	LVEDD	LVPWT	LVESD	AORD	LVEF	RWT	LV mass	LVMI	baPWV
Duration	*r*	0.134**	0.034	0.031	0.014	0.047	0.110**	−0.060	0.012	0.031	0.081*	0.316**
	*p*	<0.001	0.359	0.399	0.712	0.206	0.003	0.107	0.757	0.403	0.029	<0.001
Age	*r*	0.269**	0.112**	0.133**	0.024	0.115**	0.196**	−0.096*	0.005	0.112**	0.260**	0.565**
	*p*	<0.001	0.002	<0.001	0.523	0.002	<0.001	0.010	0.888	0.003	<0.001	<0.001
Hypertension history	*r*	0.274**	0.167**	0.178**	0.119**	0.156**	0.099**	−0.053	0.064	0.203**	0.239**	0.390**
	*p*	<0.001	<0.001	<0.001	0.001	<0.001	0.008	0.157	0.088	<0.001	<0.001	<0.001
DBP	*r*	0.076*	−0.037	0.110**	0.032	0.067	0.080*	0.031	−0.065	0.044	−0.016	0.120**
	*p*	0.040	0.321	0.003	0.397	0.074	0.032	0.408	0.080	0.235	0.661	0.001
SBP	*r*	0.230**	0.109**	0.234**	0.099**	0.157**	0.115**	0.001	−0.012	0.195**	0.216**	0.439**
	*p*	<0.001	0.003	<0.001	0.008	<0.001	0.002	0.974	0.748	<0.001	<0.001	<0.001
GLP‐1RA	*r*	0.148**	0.041	0.159**	0.043	0.116**	0.111**	−0.027	−0.077*	0.116**	0.068	−0.109**
	*p*	<0.001	0.274	<0.001	0.243	0.002	0.003	0.465	0.039	0.002	0.070	0.003
DPP‐4i	*r*	0.198**	0.144**	0.134**	0.148**	0.106**	0.080*	−0.014	0.046	0.182**	0.196**	0.039
	*p*	<0.001	<0.001	<0.001	<0.001	0.004	0.031	0.708	0.218	<0.001	<0.001	0.292
Glucosidase inhibitors	*r*	0.024	0.002	0.007	−0.008	−0.002	0.019	−0.024	0.000	−0.013	−0.006	−0.098**
	*p*	0.521	0.960	0.853	0.839	0.957	0.614	0.524	0.996	0.737	0.879	0.009
Thiazolidinediones	*r*	−0.290**	−0.161**	−0.270**	−0.164**	−0.211**	−0.148**	0.040	0.027	−0.277**	−0.243**	0.021
	*p*	<0.001	<0.001	<0.001	<0.001	<0.001	<0.001	0.279	0.473	<0.001	<0.001	0.575
SGLT‐2i	*r*	0.059	0.046	−0.016	0.030	−0.001	0.059	−0.011	0.034	0.008	0.063	0.244**
	*p*	0.112	0.217	0.659	0.429	0.989	0.113	0.759	0.358	0.830	0.091	<0.001
Sulfonylureas/nateglinide/repaglinide	*r*	−0.033	−0.024	−0.054	−0.025	−0.087*	−0.037	−0.008	0.019	−0.050	−0.023	−0.006
	*p*	0.380	0.525	0.147	0.501	0.019	0.319	0.831	0.614	0.179	0.539	0.879
Biguanides	*r*	0.201**	0.087*	0.139**	0.071	0.107**	0.123**	0.044	−0.017	0.145**	0.118**	−0.003
	*p*	<0.001	0.020	<0.001	0.056	0.004	0.001	0.234	0.654	<0.001	0.002	0.945
Insulin	*r*	−0.174**	−0.123**	−0.157**	−0.118**	−0.111**	−0.102**	−0.025	−0.006	−0.175**	−0.168**	0.000
	*p*	<0.001	0.001	<0.001	0.002	0.003	0.006	0.506	0.862	<0.001	<0.001	0.992
CCBs	*r*	0.261**	0.196**	0.169**	0.191**	0.177**	0.131**	−0.060	0.062	0.248**	0.277**	0.360**
	*p*	<0.001	<0.001	<0.001	<0.001	<0.001	<0.001	0.107	0.098	<0.001	<0.001	<0.001
ACEI/ARBs	*r*	0.101**	0.005	−0.039	−0.002	−0.014	0.002	0.020	0.027	−0.017	−0.071	0.240**
	*p*	0.006	0.902	0.299	0.962	0.701	0.958	0.588	0.470	0.653	0.056	<0.001
Antiplatelet medications	*r*	−0.056	0.002	−0.011	0.016	−0.010	−0.024	−0.025	0.030	0.007	0.008	−0.014
	*p*	0.136	0.961	0.772	0.665	0.790	0.525	0.497	0.414	0.854	0.840	0.706
Lipid‐lowering medications	*r*	−0.007	−0.007	0.024	−0.021	0.017	−0.024	0.030	−0.036	0.002	0.035	0.055
	*p*	0.845	0.847	0.513	0.571	0.651	0.514	0.423	0.338	0.958	0.345	0.142
BMI	*r*	0.357**	0.149**	0.294**	0.163**	0.226**	0.158**	−0.057	0.010	0.277**	0.234**	−0.096**
	*p*	<0.001	<0.001	<0.001	<0.001	<0.001	<0.001	0.129	0.784	<0.001	<0.001	0.010
NC	*r*	0.225**	0.102**	0.145**	0.062	0.125**	0.110**	−0.042	0.010	0.135**	0.065	−0.066
	*p*	<0.001	0.006	<0.001	0.094	0.001	0.003	0.260	0.797	<0.001	0.080	0.077
WC	*r*	0.383**	0.139**	0.268**	0.153**	0.225**	0.170**	−0.068	0.014	0.258**	0.179**	0.004
	*p*	<0.001	<0.001	<0.001	<0.001	<0.001	<0.001	0.066	0.708	<0.001	<0.001	0.920
WHR	*r*	0.314**	0.115**	0.173**	0.073*	0.168**	0.097**	−0.052	0.009	0.162**	0.170**	0.117
	*p*	<0.001	0.002	<0.001	0.049	<0.001	0.010	0.165	0.803	<0.001	<0.001	0.002
VFA	*r*	0.323**	0.172**	0.253**	0.140**	0.195**	0.134**	−0.102**	0.029	0.258**	0.230**	0.083*
	*p*	<0.001	<0.001	<0.001	<0.001	<0.001	<0.001	0.006	0.441	<0.001	<0.001	0.027
SFA	*r*	0.300**	0.099**	0.203**	0.141**	0.165**	0.113**	−0.037	0.019	0.202**	0.120**	−0.061
	*p*	<0.001	0.008	<0.001	<0.001	<0.001	0.002	0.318	0.602	<0.001	0.001	0.103
VSR	*r*	0.003	0.055	0.029	−0.037	0.011	−0.004	−0.076*	−0.010	0.024	0.060	0.143**
	*p*	0.933	0.139	0.432	0.320	0.770	0.922	0.040	0.784	0.515	0.108	<0.001
VFA/BMI	*r*	0.230**	0.139**	0.163**	0.089*	0.127**	0.088*	−0.101**	0.031	0.177**	0.121**	0.163**
	*p*	<0.001	<0.001	<0.001	0.017	0.001	0.018	0.006	0.405	<0.001	0.001	<0.001
VFA/H2	*r*	0.310**	0.170**	0.222**	0.116**	0.171**	0.110**	−0.103**	0.031	0.231**	0.263**	0.130**
	*p*	<0.001	<0.001	<0.001	0.002	<0.001	0.003	0.006	0.410	<0.001	<0.001	<0.001
VFA/W	*r*	0.208**	0.134**	0.121**	0.058	0.097**	0.058	−0.099**	0.034	0.141**	0.207**	0.231**
	*p*	<0.001	<0.001	0.001	0.121	0.009	0.121	0.008	0.363	<0.001	<0.001	<0.001
FPG	*r*	0.035	0.018	0.014	−0.002	−0.011	0.018	0.001	0.003	0.018	0.022	0.094*
	*p*	0.351	0.623	0.702	0.947	0.762	0.634	0.981	0.934	0.626	0.562	0.012
FIns	*r*	−0.012	−0.050	−0.005	−0.049	0.010	−0.035	−0.013	−0.039	−0.045	−0.055	0.055
	*p*	0.785	0.240	0.907	0.246	0.807	0.412	0.763	0.361	0.287	0.196	0.200
FC‐P	*r*	0.178**	0.061	0.095*	0.030	0.112**	0.086*	−0.064	−0.001	0.077*	0.031	−0.008
	*p*	<0.001	0.103	0.010	0.422	0.003	0.021	0.086	0.968	0.039	0.413	0.836
HbA1c	*r*	−0.041	−0.014	−0.056	0.065	−0.046	−0.038	0.020	0.066	0.004	−0.004	0.032
	*p*	0.272	0.701	0.135	0.079	0.218	0.310	0.585	0.076	0.917	0.912	0.397
GA	*r*	−0.056	−0.015	−0.073*	0.033	−0.057	−0.006	0.048	0.065	−0.016	−0.004	0.047
	*p*	0.132	0.682	0.050	0.377	0.131	0.865	0.199	0.082	0.674	0.920	0.211
HOMA‐IR	*r*	0.173**	0.054	0.091*	0.014	0.094*	0.073*	−0.059	−0.010	0.067	0.029	0.065
	*p*	<0.001	0.147	0.015	0.712	0.011	0.049	0.112	0.782	0.070	0.433	0.083
TC	*r*	0.006	0.033	−0.055	0.011	−0.041	−0.012	−0.021	0.053	−0.007	−0.008	0.011
	*p*	0.873	0.375	0.142	0.775	0.273	0.748	0.573	0.159	0.843	0.840	0.779
TG	*r*	0.081*	0.005	0.001	0.020	−0.002	0.015	0.014	0.014	0.005	−0.037	−0.035
	*p*	0.031	0.904	0.976	0.586	0.953	0.683	0.701	0.714	0.884	0.329	0.347
HDL‐c	*r*	−0.058	−0.006	−0.008	−0.028	−0.028	−0.020	0.013	−0.020	−0.019	0.031	0.089*
	*p*	0.127	0.869	0.829	0.461	0.451	0.604	0.726	0.595	0.609	0.404	0.018
LDL‐c	*r*	−0.024	0.024	−0.057	−0.007	−0.049	−0.016	−0.031	0.038	−0.018	−0.019	−0.021
	*p*	0.521	0.530	0.129	0.843	0.194	0.662	0.411	0.316	0.630	0.613	0.584
UA	*r*	0.112**	0.076*	−0.008	0.009	0.089*	0.085*	−0.006	0.070	0.028	−0.006	−0.010
	*p*	0.003	0.041	0.832	0.815	0.017	0.024	0.869	0.063	0.453	0.880	0.794
BUN	*r*	0.114**	0.082*	0.067	0.039	0.079*	0.080*	−0.117**	0.034	0.082*	0.118**	0.222**
	*p*	0.002	0.028	0.075	0.301	0.035	0.034	0.002	0.368	0.029	0.002	<0.001
Cr	*r*	0.066	0.078*	0.025	0.018	0.031	0.041	−0.055	0.047	0.055	0.078*	0.172**
	*p*	0.079	0.037	0.506	0.628	0.415	0.273	0.143	0.214	0.144	0.037	<0.001
e‐GFR	*r*	0.002	−0.028	0.077*	0.054	0.034	−0.044	0.071	−0.032	0.052	−0.104**	−0.377**
	*p*	0.956	0.457	0.040	0.149	0.367	0.244	0.059	0.386	0.163	0.005	<0.001
baPWV	*r*	0.222**	0.128**	0.117**	0.073	0.111**	0.152**	−0.062	0.05	0.135**	0.236**	/
	*p*	<0.001	0.001	0.002	0.052	0.003	<0.001	0.100	0.179	<0.001	<0.001	/

Abbreviations: ACEI/ARBs, angiotensin‐converting enzyme inhibitors/angiotensin II receptor blocker; AORD, aortic root inside diameter; baPWV, brachial‐ankle pulse wave velocity; BMI, body mass index; BUN, blood urea nitrogen; CCBs, calcium channel blockers; DBP, diastolic blood pressure; DM, diabetes mellitus; DPP‐4i, dipeptidyl peptidase‐4 inhibitor; e‐GFR, estimated glomerular filtration rate; FC‐P, fasting C‐peptide; FIns, fasting insulin; FPG, fasting plama glucose; GA, glycated albumin; GLP‐1RA, glucagon‐like peptide‐1 receptor agonist; HbA1c, glycosylated hemoglobin; HDL‐C, high‐density cholesterol; HOMA‐IR, homeostasis model assessment of insulin resistance; IVST, interventricular septal thickness; LAD, left atrial diameter; LDL‐C, low‐density cholesterol; LV mass, left ventricular mass; LVEDD, left ventricular end‐diastolic diameter; LVEF, left ventricular ejection fraction; LVESD, left ventricular end‐systolic diameter; LVMI, left ventricular mass index; LVPWT, left ventricular posterior wall thickness; NC, neck circumference; RWT, relative wall thickness; SBP, systolic blood pressure; Scr, serum creatinine; SFA, subcutaneous fat area; SGLT‐2i, sodium‐glucose cotransporter‐2 inhibitor; TC, total cholesterol; TG, triglycerides; UA, uric acid; VFA, visceral fat area; VSR, visceral‐to‐subcutaneous fat ratio; WC, waist circumference; WHR, waist‐to‐hip ratio.

* *p* < 0.05;

** *p* < 0.01.

We analyzed the associations of age with duration of diabetes, VFA, BMI, and WHR, and the results showed that BMI (*r* = − 0.253, *p* < 0.001), VFA (*r* = − 0.113, *p* < 0.001), and SFA (*r* = − 0.231, *p* < 0.001) in male and BMI (*r* = − 0.176, *p* < 0.001) and SFA(*r* = −0.084, *p* = 0.024) in female significantly decreased with age, while WHR (*r* = 0.106, *p* = 0.004) in female was positively associated with age. The duration of diabetes was positively associated with age in both male (*r* = 0.417, *p* < 0.001) and female (*r* = 0.381, *p* < 0.001). No associations of WHR with age in male (*r* = − 0.014, *p* = 0.599) or VFA (*r* < 0.001, *p* = 0.991) with age with female were observed. VFA/BMI increased with age in female (*r* = 0.109, *p* = 0.003) but did not change in male (*r* = 0.034, *p* = 0.203) (Table 5).

**TABLE 5:** Correlation of age with adipose deposition and DM duration.

			BMI	WHR	VFA	SFA	Duration of diabetes	VFA/BMI
Age	Male	*r*	−0.253**	−0.014	−0.113**	−0.231**	0.417**	−0.034
		*p*	<0.001	0.599	<0.001	<0.001	<0.001	0.203
	Female	*r*	−0.176**	0.106**	<0.001	−0.084*	0.381**	0.109**
		*p*	<0.001	0.004	0.991	0.024	<0.001	0.003
Duration of diabetes	Male	*r*	−0.143**	0.017	−0.111**	−0.105**	1.000	−0.081**
		*p*	<0.001	0.517	<0.001	<0.001	/	0.002
	Female	*r*	−0.093*	0.134**	−0.024	−0.071	1.000	0.018
		*p*	0.012	<0.001	0.515	0.056	/	0.637

Abbreviations: BMI, body mass index; SFA, subcutaneous fat area; VFA, visceral fat area; WHR, waist‐to‐hip ratio.

* *p* < 0.05;

** *p* < 0.01.

### 3.3 Multivariable analysis

Multivariate linear regression analysis was carried out to assess the independent covariates of LVEF, LVMI, and baPWV (Tables 6, 7, 8) after adjusting for confounding factors such as age, duration of diabetes, hypertension history, and medications. As shown in Table 6, in male patients, levels of HbA1c (*β* = − 0.120, *p* < 0.001), WHR (*β* = − 0.078, *p* = 0.011), and FC‐P (*β* = − 0. 068, *p* = 0.030) were significantly associated with LVEF, whereas in female patients, BUN (*β* = − 0.115, *p* < 0.001) and VFA (*β* = − 0.096, *p* = 0.010) were significantly associated with LVEF.

**TABLE 6:** Multivariate linear regression analysis for independent factors associated with LVEF in male and female patients with type 2 diabetes.

Model		B	SE	*β*	*t*	*p*
Male^a^	HbA1c	−0.144	0.033	−0.120	−4.301	<0.001
	WHR	−0.023	0.009	−0.078	−2.537	0.011
	FC‐P	−0.001	0.000	−0.068	−2.173	0.030
Female^b^	BUN	−0.187	0.060	−0.115	−3.117	0.002
	VFA	−0.007	0.003	−0.096	−2.588	0.010

Abbreviations: BUN, blood urea nitrogen; FC‐P, fasting C‐peptide; HbA1c, glycosylated hemoglobin; VFA, visceral fat area; WHR, waist‐to‐hip ratio.

^a^ Adjusted for age, hypertension history, CCBs, GLP‐1RA, lipid‐lowering medications, variables included in the model were BMI, WHR, GA, FPG, HbA1c, FC‐P, baPWV, Cr.

^b^ Adjusted for age and CCBs, variables included in the model included age, BUN, and VFA.

**TABLE 7:** Multivariate linear regression analysis for independent factors associated with LVMI in male and female patients with type 2 diabetes.

Model		*B*	SE	*β*	*t*	*p*
Male^a^	BMI	0.012	0.002	0.242	4.691	<0.001
	baPWV	<0.001	0.000	0.109	3.997	<0.001
	SFA	0.000	0.000	−0.155	−3.033	0.002
Female^b^	WHR	11.020	3.956	0.101	2.786	0.005
	baPWV	0.002	0.001	0.103	2.459	0.014
	VFA	0.019	0.009	0.083	2.074	0.038

Abbreviations: baPWV, brachial‐ankle pulse wave velocity; BMI, body mass index; SFA, subcutaneous fat area; VFA, visceral fat area; WHR, waist‐to‐hip ratio.

^a^ Adjusted for age, duration of diabetes, hypertension history, GLP‐1RA, DPP‐4i, glucosidase inhibitors, thiazolidinediones, insulin, CCBs, ACEI/ARBs, antiplatelet medications, lipid‐lowering medications, SGLT‐2i, variables included in the model were DBP, SBP, BMI, NC, WC, WHR, VFA, SFA, BUN, Cr, e‐GFR, baPWV, FC‐P.

^b^ Adjusted for duration of diabetes, age, hypertension history, DPP4, thiazolidinediones, biguanides, CCBs, insulin, variables included in the model were baPWV, WC, WHR, VFA, SFA, BUN, Cr, and e‐GFR.

**TABLE 8:** Multivariate linear regression analysis for independent factors associated with baPWV in male and female patients with type 2 diabetes.

Model		*B*	SE	*β*	*t*	*p*
Male^a^	SBP	4.643	0.574	0.254	8.094	<0.001
	DBP	3.319	0.902	0.115	3.678	<0.001
	e‐GFR	−0.799	0.187	−0.126	−4.267	<0.001
	WHR	299.445	135.238	0.049	2.214	0.027
	FPG	6.014	2.398	0.054	2.508	0.012
Female^b^	SBP	6.817	0.605	0.33	11.263	<0.001
	FPG	13.204	3.836	0.095	3.442	0.001
	BMI	−16.431	4.046	−0.170	−4.061	<0.001
	VFA	1.329	0.450	0.120	2.951	0.003

Abbreviations: BMI, body mass index; DBP, diastolic blood pressure; e‐GFR, estimated glomerular filtration rate; FPG, fasting plama glucose; SBP, systolic blood pressure; Scr, serum creatinine; VFA, visceral fat area; WHR, waist‐to‐hip ratio.

^a^ Adjusted for age, duration of diabetes, hypertension history, glucosidase inhibitors, thiazolidinediones, insulin, CCBs, ACEI/ARBs, lipid‐lowering medications, variables included in the model were DBP, SBP, WHR, VSR, FPG, HbA1c, TC, HDL‐C, LDL‐C, and e‐GFR.

^b^ Adjusted for duration of diabetes, age, hypertension history, GLP‐1RA, glucosidase inhibitors, SGLT‐2i, CCBs, ACEI/ARBs, variables included in the model were SBP, DBP, BMI, WHR, FPG, HDL‐C, BUN, Cr, e‐GFR, VFA.

In Table 7, LVMI was significantly associated with baPWV (*β* = 0.109, *p* < 0.001), SFA (*β* = − 0.155, *p* = 0.002), and BMI (*β* = 0.242, *p* < 0.001) in male patients, whereas in female patients, LVMI was significantly associated with VFA (*β* = 0.083, *p* = 0.038), baPWV (*β* = 0.103, *p* = 0.014), and WHR (*β* = 0.101, *p* = 0.005).

As for baPWV, multivariable‐adjusted linear regression models showed that SBP and FPG were independently associated with baPWV in both sexes. DBP (*β* = 0.115, *p* < 0.001), e‐GFR (*β* = − 0.126, *p* < 0.001), and WHR (*β* = 0.049, *p* = 0.027) were associated with baPWV in male patients. Simultaneously, baPWV was observed to be significantly associated with BMI (*β* = − 0.170, *p* < 0.001) and VFA (*β* = 0.120, *p* = 0.003) in females, respectively (Table 8).

## 4 DISCUSSION

To our knowledge, there is a scarcity of epidemiological data that specifically establish a connection between VFA and the correlation of cardiac hemodynamics and arterial stiffness (baPWV) in individuals of different sexes with diabetes. In the current study, we conducted an examination of the disparities in visceral adipose tissue, LV function and deformation, and arterial stiffness (baPWV) in patients with diabetes with normal LVEF (LVEF ≥ 50%). The primary findings of this cross‐sectional study are as follows: (1) Male patients exhibited higher levels of VFA and proportion of VFA (+) than female ones. (2) Males displayed lower values of LVEF and LVMI in comparison with females. (3) Both men and women demonstrated deteriorated parameters of metabolic status parameters and enlarged LV structure in the highest quartile of VFA. (4) VFA was independently associated with LVEF, LVMI, and baPWV only in females. (5) Levels of baPWV were higher in women than in men, and increased in the Q4 of VFA. baPWV was positively associated with LVMI in both men and women, but negatively associated with LVEF only in men.

### 4.1 Fat distribution in different sexes

Our study showed that levels of VFA and the proportion of increased VFA (VFA > 100 cm^2^) were higher, whereas SFA and VFA/weight were lower in male patients than in females. As body fat distribution differed between men and women,^25^ men have lower percentages of total body fat but are prone to abdominal adiposity than women; sex‐specific relationship of VFA with cardiac hemodynamics and atherosclerosis using sex subgroup analysis is essential. A cross‐sectional study^26^ has revealed that VFA and WHR increase significantly with age in both sexes among Beijing adults aged 20–60 years. In our study, BMI decreased with age and duration of diabetes in both male and female patients. However, VFA significantly decreased with age and duration of diabetes only in males but not in females. We speculate that diabetes management that affect body weight may cause the decreasing of BMI in both sexes. Results of Gao et al.^27^ showed that comparing with women aged in 50–65 years old, females over 65 years old had higher VFA, but not higher BMI. Previous studies^28^, ^29^ have shown that estrogen deficiency may result in visceral fat accumulation and that postmenopausal women are prone to central obesity, metabolic syndrome, type 2 diabetes, and cardiovascular diseases. Our study is partly consistent with their results, that VFA/BMI but not VFA increased with age in female, indicating that the age/hormone‐related muscle loss^30^ and relative increase of VFA may be more apparent in female patients.

### 4.2 Cardiac hemodynamics in different sexes

Previous studies have demonstrated^31^, ^32^ that there is a greater increase in the incidence of heart failure (HF) among men. It was also demonstrated that age, male sex, hypertension, LV hypertrophy, myocardial infarction, valvular heart disease, obesity, and diabetes are major clinical risk factors of HF in general population.^33^ Our current study indicated that values of LVEF and LVMI were higher in women than in men. As the LVEF was negatively associated with age in our study (*r* = − 0.096, *p* < 0.001 in men, *r* = − 0.096, *p* = 0.010 in women), we found that despite the younger age, men's levels of LVEF seem more likely to be affected by diabetes. Increasing of LVMI can result in left ventricular hypertrophy (LVH), which is an important predictor of CVD.^34^ Female patients' higher values of LVMI in the current study were partly due to the fact that they were older than males and had a longer duration of diabetes. A cohort study which enrolled 7487 patients indicated^35^ that women with HF exhibited a significantly higher mean age and left ventricular ejection fraction than men. Results of follow‐up^35^ showed that cumulative incidence of death in men with ischemic cardiomyopathy (iCMP) were higher, that in which study, diabetes was one of the main predictors of death for iCMP in women and men. Another cross‐sectional study in Yunnan, China,^36^ also showed a relatively higher ejection fraction in females than in males. The Journey Heart Failure‐Turkish Population study demonstrated that female patients with HF had better LVEF but higher in‐hospital mortality than males, indicating that LVEF is not a dominant predictor of HF prognosis in females. It has been recognized that^37^ females have smaller body size‐adjusted cardiac volumes and more easily to suffer from age‐related myocardial hypertrophy. The higher LVEF and LVMI in females indicated that LVEF can be more preserved in female patients, but the LVMI may be a more sensitive indicator for subclinical changes of cardiac remodeling in patients with DM.

### 4.3 Correlations of VFA and cardiac hemodynamics characteristics

Naoko Sawada et al.^38^ revealed that VFA was significantly associated with decreased left ventricular global longitudinal strain (LVGLS) and right ventricular free‐wall longitudinal strain (RVLS), which might be involved in the increased risk of HF in obese individuals. By observing that women with T2DM have a different fat distribution profile from that of men, and women show lower VFA and higher SFA comparing with men, we speculate the varied predisposition to cardiac dysfunction influenced by visceral fat accumulation between men and women can be existed. In patients with diabetes,^39^ VFA was reported to be an independent determinant of LVMI, LVEF, and left atrial diameter, and predicted cardiovascular disease risk. A recent study^40^ has demonstrated that obesity dominates early effects on cardiac structure in people with type 2 diabetes. It is also reported that^41^ increased VFA is an independent risk factor for atherosclerosis in female patients with normal body weight. In this current study, both men and women demonstrated deteriorated parameters of metabolic status parameters and enlarged LV structure, as well as lower LVEF, in the highest quartile of VFA. However, we found a correlation of VFA with LVEF only in female patients (Table 4, Figure 2), indicating that increasing VFA can predict lower LVEF only in women. In the Q4 group of VFA, females presented the lowest LVEF and highest baPWV among the four quartiles, and significantly different from the Q1 group (*p* < 0.05). On the other hand, LVMI tended to increase according to increasing VFA quartiles in women; whereas in men, LVMI did not significantly increase from Q1 to Q3, but increased in Q4. Although Spearman correlation analysis showed that LVMI were associated with VFA in both males and females, results of multivariate linear regression analysis indicated that VFA was independent associated with LVEF and LVMI only in women. This suggests that female patients' cardiac structure and hemodynamics might be more susceptible to the increasing of visceral fat. Our study also showed that in male patients, SFA was an independent indicator of LVMI, which was inconsistent with the results of Qiu et al.^39^ The inconsistence may be result from the study population, different ages patients recruited, and different diabetes management. Additionally, although there were several clinical variables significantly correlated with LVEF, the correlations seemed relatively weak (*r* < 0.2). We speculate that as patients with LVEF<50% were excluded from the subjects, LVEF levels of participants in our study were within a narrow range (50%–72%). This might result in a significantly association accompanied with a low “*r*” value. Also, VFA was one of the most significantly correlated variables with LVEF in female group, while no significant correlations were found between LVEF and VFA in the male group, despite its larger number of participants.

### 4.4 Correlations of VFA and baPWV

Significant differences in arterial stiffness between women and men by age was suggested in a growing body of evidence.^42^, ^43^ Abdominal adiposity was considered to be more associated with arterial stiffness in younger women than in men, indicating a potentially greater effect of concentric obesity in increasing CVD risk in women than in men. To examine the sex‐specific relationship between VFA and arterial stiffness in patients with diabetes, sex subgroup analysis was carried out. Recently, Xu et al.^41^ have investigated the association of VFA and baPWV, and suggested that VFA was significantly associated with tertiles of baPWV in female type 2 diabetes patients with normal weight, whereas no statistical significance was obtained among male type 2 diabetes patients. As shown in Table 1 in our study, baPWV in men were lower than those in women. In agreement with other studies,^16^, ^17^, ^18^ baPWV is positively associated with age in this current study. We speculated that the reason for difference of baPWV between men and women might be that male patients were younger than female patients in this study. We also found that VFA was independently correlated with baPWV in female patients. However, we did not find any association of VFA with baPWV in male patients. These results were in accordant with results of Xu et al.^41^ that the adverse effects of VFA on baPWV were stronger in women than in men.

### 4.5 Limitations

There are several limitations to the current study. First, significant differences were found in age and duration of DM between the male patients and females. These may have some effects on the differences between two groups, which may also result in significantly differences in medication use. Female patients were older and had a longer duration of diabetes, higher proportion of hypertension, which may increase the possibility of using of insulin, CCBs, ACEI/ARBs in these patients. Second, as a cross‐sectional single‐center study, there may be some bias in the results and the results in the present study cannot establish causality. Medications would probably affect patients' VFA and cardiovascular outcomes. In order to minimize the bias, we adjusted for age, duration of diabetes, history of hypertension, medications in the regression models. Further longitudinal follow‐up studies are needed to verify if different levels of baseline VFA and its change could influence cardiac hemodynamics in T2DM after adjusting the influence of medications. Besides, the VFA values were derived from bioelectrical impedance analysis (BIA) instead of CT scans, as BIA is a more widely available, low‐cost, and non‐x‐ray‐based method. Additionally, although all the enrolled patients were able to take care of themselves, not all of them could clearly describe the time and intensity of their physical activities (PA) during the last 7 days before the enrollment. So, we were unable to analyze the correlations of VFA and cardiac hemodynamics with their PA levels. We have collected those who can calculate their quantity of exercise into the database using the International Physical Activity Questionnaire‐Short Form (IPAQ‐SF) Chinese edition and started a long‐term follow‐up, so that we can see the future change of cardiac hemodynamics and their potential associations with PA levels.

## 5 CONCLUSIONS

In conclusion, our study suggests sex differences for visceral fat obesity and cardiac hemodynamic parameters, that VFA were independently associated with LVEF, LVMI, and baPWV in women, but not in men, in patients with diabetes. Our results suggested that VFA is significantly associated with changes of left ventricular function and deformation in female patients with T2DM. More attention should be paid to the relationship between central obesity, cardiac structure, and vascular disease in female T2DM patients in clinical practice.

## AUTHOR CONTRIBUTIONS

P.Y and W.Y designed the study and reviewed and edited the manuscript. C.R and L.Y wrote and edited the manuscript, and researched data. C.R, L.Y, and X.H researched data.

## FUNDING INFORMATION

This work was supported by DMRFP_2_12 from Diabetes Mellitus Research Fund Program from Shanghai Medical and Health Development Foundation (SHMHDF), National Key Research and Development Program of China (2021YFC2501600, 2021YFC2501601), and Shanghai science and Technology Innovation Fund (21Y11904800).

## CONFLICT OF INTEREST STATEMENT

The authors declared no potential conflicts of interest with respect to the research, authorship, and/or publication of this article.
